# Parity moderates the effect of delivery mode on maternal ratings of infant temperament

**DOI:** 10.1371/journal.pone.0255367

**Published:** 2021-08-12

**Authors:** Lea Takács, Samuel P. Putnam, František Bartoš, Pavel Čepický, Catherine Monk

**Affiliations:** 1 Department of Psychology, Faculty of Arts, Charles University, Prague, Czech Republic; 2 Department of Psychology, Bowdoin College, Brunswick, Maine, United States of America; 3 Department of Obstetrics & Gynecology, and Psychiatry, Columbia University Irving Medical Center, New York, New York, United States of America; 4 New York State Psychiatric Institute, New York, New York, United States of America; Wayne State University, UNITED STATES

## Abstract

**Objective:**

Cesarean section (CS) rates are rising rapidly around the world but no conclusive evidence has been obtained about the possible short- and long-term effects of CS on child behavior. We evaluated prospectively the association between CS and infant temperament across the first 9 postpartum months, controlling for indications for CS and investigating parity and infant sex as moderators.

**Methods:**

The sample consisted of mothers and their healthy infants. Infant temperament was measured using the Infant Characteristics Questionnaire completed by the mothers at 6 weeks (n = 452) and 9 months (n = 258) postpartum. Mode of birth was classified into spontaneous vaginal birth (n = 347 for 6 weeks sample; 197 for 9 months sample), CS planned for medical reasons (n = 55; 28) and emergency CS (n = 50; 33).

**Results:**

Multiple regression analysis revealed no main effects of birth mode, but showed a significant interaction between birth mode and parity indicating that emergency CS in firstborn infants was associated with more difficult temperament at 6 weeks. There were no significant associations between indications for CS and infant temperament, although breech presentation predicted difficult temperament at 9 months.

**Conclusion:**

We largely failed to support the association between CS and infant temperament. Although our results suggest that emergency CS may be associated with temperament in firstborns, further research is needed to replicate this finding, preferably using observational measures to assess child temperament.

## Introduction

Worldwide, 19% of births occur by cesarean section (CS), with the highest rates found in more developed countries [1]. Between 1990 and 2014, the CS rate increased from 11% to 25% in Europe and from 22.3% to 32.3% in North America [1]. While CS may be a life-saving intervention in cases of pregnancy or childbirth complications, it is also associated with reproductive health risks. A recent systematic review showed that women who undergo CS are at a higher risk of miscarriage, stillbirth, placenta previa, placenta accreta, and placental abruption in future pregnancies [2].

Risks associated with CS may extend to children as well, possibly because CS is often conducted due to emergency indications and health complications which themselves may present challenges (e.g., fetal distress, preeclampsia). Children born by CS have been found to have an increased risk of asthma, obesity, and type-1 diabetes [3]. Furthermore, birth by CS has been associated with risks for neurodevelopmental disorders such as autism and attention-deficit/hyperactivity disorder [4], although recent evidence suggest that those associations are most likely due to unmeasured familial confounding [5]. Some studies reported delays in cognitive and motor development in children born by CS, but the results of these studies are inconsistent [6, 7].

Moreover, some existing research suggests that CS may affect child temperament. Although early perspectives on temperament [8, 9] emphasized the genetic underpinnings of early appearing traits, contemporary perspectives acknowledge the influence of both prenatal and postnatal environments on the hormonal and neural systems associated with characteristic patterns of emotional response [10, 11]. To date, only a few studies have focused on the association between CS and infant temperament. Field and Widmayer [12] observed that mothers who gave birth per emergency CS, compared to those who gave birth vaginally, rated their infants as having more optimal temperament 4 and 8 months postpartum. Maziade et al. [13], on the other hand, found no relation between birth mode and infant temperament at these same ages. Simons et al. [14] showed that infants born through CS versus vaginal birth were rated by their parents as less adaptable. In addition, examining the interaction between birth mode and infant risk status, Simons et al. [14] observed that CS-born infants in a low-risk group were perceived as more withdrawing, more negative, and less adaptable than those from high-risk and/or vaginal birth groups. The authors of this study did not, however, indicate whether their results applied to emergency or planned CS.

Related research on the associations between CS and child emotional and behavioral problems has likewise yielded conflicting results. Some studies reported adverse effects of CS on child outcomes, such as a higher risk of parent-reported internalizing problems (e.g., anxiety/depression) at 1.5 and 5 years of age [15, 16] and increased stress behavior two hours after birth [17], while others found no effect of CS on child behavior reported by parents [6, 18–20]. One large study [21] even reported that, in children aged 4–6 years, the risk of externalizing problems (e.g., aggression) was lower in those children who were born by elective CS based on maternal request rather than by spontaneous or assisted vaginal birth.

Some investigators attempted to explain the inconsistencies in existing research by identifying specific groups of children particularly vulnerable to the negative effects of CS. As noted above, Simons et al. [14] reported specific effects of CS for low-risk but not high-risk pregnancies. Several studies examined potential moderating effects of child’s sex but found no evidence for such interactions [6, 16, 19]. Although often included as a covariate or a controlled variable in the studies, parity is a hitherto overlooked possible moderator of birth practices. Since research suggests that CS has negative impact on maternal well-being (such as lower self-esteem or higher risk of post-traumatic stress symptoms) especially in primiparous women [22, 23], who are also at a higher risk of emergency CS [24], some authors speculated that the effect of birth mode on infant behavior might differ depending on parity [25]. Primiparous women have been found to report lower maternal self-efficacy [26, 27], worse mother-infant bonding [28] and higher postpartum anxiety [26] compared to multiparous women, which could, combined with their higher risk of negative psychosocial consequences of CS, lead to their tendency to perceive their child behavior as more problematic. However, to the best of our knowledge, to date no study has tested this hypothesis.

The inconsistency in existing research findings regarding the potential effects of CS on child behavioral outcomes may also be due to differing methodology. While some studies focused on maternally requested CS specifically [16, 21], others differentiated between elective and emergency CS [6, 19], and several studies failed to distinguish between different types of CS [14, 15]. Although several studies controlled for pregnancy and intrapartum complications, no study considered specific indications for CS, which might be the actual cause of children’s behavioral problems. Another gap in the previous research is that child outcomes are mostly assessed on a one-time basis later in childhood, thus yielding no information about the stability of potential effects.

Studying perinatal influences on infant temperament is of great clinical relevance as a large body of research implicates temperament as a risk factor for later behavior problems and psychopathology, with negative mood forecasting both internalizing and externalizing problems [29, 30]. The underlying mechanisms through which CS may affect child behavioral and temperamental characteristics are not yet determined, but several hypotheses have been proposed, including altered microbiota in the infant gut [31–33], programming of hypothalamic–pituitary–adrenal (HPA) axis functioning [17, 34, 35], and “iatrogenic prematurity” in infants born before optimal maturation of the fetal brain [36].

The effects of CS may additionally be mediated by elements of the infant’s social context following birth. An early meta-analysis of relations between delivery mode and psychosocial outcomes [37] indicated that, in comparison to mothers who had delivered vaginally, mothers who underwent CS experienced less satisfaction with the birth, responded less positively to their newborns, were less likely to breastfeed, and interacted less with their infants in the home. More recent studies continue to support these findings [38, 39], and it has been proposed that increased risk for postpartum depression, changes in neural responses to infant crying, and feelings of failure among mothers who have undergone CS, may affect their maternal behaviors [22, 40, 41]. Indeed, CS has been associated with parenting that is characterized by less stimulation, caretaking and intimate play [42]. Because maternal depression, self-efficacy, and parenting behavior have been related to difficult temperament [43, 44]; and because increased levels of infant negativity and fear have been related to low maternal responsivity and sensitivity [45–47]; it is reasonable to expect CS to affect infant difficulty through its effect on mothers’ psychology and actions. A final mechanism potentially relating CS to temperament also concerns maternal psychology. Feelings of disappointment, diminished self-efficacy and depression are associated with cognitive biases, including increased attention to perception of negative information [48–50]. As previous studies operationalize temperament and behavioral features through parent report questionnaires, maternal psychology associated with CS may result in higher ratings of difficulty by biasing the reports of mothers, causing them to overestimate the degree to which their child exhibits characteristics associated with difficulty [14–16].

In this study, we aimed to obtain a more complete picture of the effects of CS than previous studies by distinguishing between emergency and planned CS, controlling for indications for CS, assessing infant temperament repeatedly (6 weeks and 9 months postpartum), and investigating parity as a moderator. We hypothesized that infants born through planned or emergency CS, versus those born vaginally, would display more difficult temperament. Furthermore, we anticipated that the effect of CS on infant temperament would be moderated by parity. In particular, we hypothesized that CS would be most strongly associated with less favorable infant temperament ratings in primiparous women. We also expected to replicate the finding of earlier studies that concluded that the effect of CS on infant behavior is not moderated by infant’s sex.

## Materials and methods

### Procedure

This study is part of a prospective longitudinal project examining perinatal determinants of child development. The sample was recruited in collaboration with five maternity hospitals in the Vysočina Region of the Czech Republic. At recruitment, women were approached by the hospital staff during an antenatal visit or their hospital stay after giving birth and invited to participate in the study. They were given oral and written information about the study and asked to sign an informed consent form if they agreed to participate.

The data used in the present study were collected via questionnaires administered at the maternity hospitals during women’s postpartum stay in the hospital (T1) and online or in a paper version sent via mail (according to woman’s choice) at 6 weeks (T2) and 9 months postpartum (T3). At T1, the participants completed a questionnaire regarding their sociodemographic background. At T2 and T3, they completed a questionnaire assessing their infant’s temperament (Infant Characteristics Questionnaire, ICQ) [51]. Data concerning the mode of birth and indications for CS were extracted from medical records, except that mothers reported on whether their elective CS was based on medical reasons or their own request. The study was approved by the Ethics Committee of the Jihlava Hospital.

### Participants

Using the already existing data, we did not conduct a power analysis to determine the sample size for this study. We used all data that were available from the existing cohort. A total of 1190 women completed the background questionnaire at T1 and had data from the medical records available. Of these women, 661 women also completed the ICQ at T2 and 392 women at T3. To make maximal use of collected data, we did not exclude women who completed the questionnaire at T3 but not at T2 (that is, the T3 sample was not created directly from the T2 sample). From T2 to T3, 294 women dropped out of the study, whereas 25 women completed the questionnaires at T3 but not at T2.

To limit possible bias related to the effects of maternal pregnancy complications and infant health problems, we excluded infants of mothers with a serious pathology during pregnancy which might have affected fetal development (diabetes = 75, hypertension = 62) as well as infants with unfavorable health outcomes (Apgar score at 5 minutes < 8 = 10, birth weight < 2,500 g = 34, gestational age at birth < 36 weeks or > 40 weeks = 152, and postpartum hospitalization > 10 days = 17). Other exclusion criteria were maternal age < 18 or > 45 years (n = 3) and multiple pregnancy (n = 13). We also excluded women who had undergone vaginal operative birth (vex, forceps) (n = 26) and those who had a planned CS based on their own request (n = 11) without medical reasons, because those groups were too small to form separate categories of birth mode for the analyses. The planned CS group thus included only women with CS planned for medical reasons which were unlikely to affect fetal development (e.g., previous CS).

After applying the exclusion criteria described above and removing mothers with missing values on medical variables, sociodemographic variables or temperament questionnaire, our sample consisted of 731 mother-infant pairs with data for T1 available, 452 mother-infant pairs with data for T1 and T2, and 258 mother-infant pairs with data for T1 and T3. A comparison between mothers who dropped out of the study from T2 to T3 and those who remained until T3 shows that the only significant difference between those two groups was that women with higher educational level were more likely to remain in the study (S1 Table).

### Measures

#### Outcome variable

We measured infant temperament using an adaptation of the Infant Characteristics Questionnaire [51]. The ICQ is a screening tool for infant difficultness measuring four temperamental dimensions: Fussy/Difficult, Unadaptable, Dull, and Unpredictable. The original version of the ICQ contains 24 items, but based on factor analysis reported by the authors [51], we applied only 16 items in this study. These items included five from the Fussy/Difficult dimension (example item: “How much does your baby cry and fuss in general”), four from Unadaptable (e.g., “How does your baby typically respond to a new person”), three from Dull (e.g., “How much does your baby smile and make happy sounds”), and four from the Unpredictable (e.g., “How consistent is your baby in sticking to his/her eating routine”) dimension. An overall difficultness score, rather than separate scores for the subdimensions, was employed, as we did not have specific hypotheses regarding the discrete temperament aspects, and in order to maximize internal consistency. Cronbach’s alpha was high for this short version of the ICQ (0.83 and 0.84 at 6 weeks and 9 months postpartum, respectively). Each item was rated on a 7-point scale, with all item scores combined to form a single score. The individual items were scored 1–7, the scores could thus range from 16 to 112, with higher scores indicating more difficult temperament. In our study, the actual score range was 20–79 for T2 and 17–69 for T3.

#### Predictor variable

The mode of birth was classified into three categories: spontaneous vaginal birth, emergency cesarean section (CS), and planned CS scheduled for medical reasons.

#### Control variables

Indications for performing CS in our sample were: fetal hypoxia, failure to progress, other labor dysfunctions (asynclitism, dystocia), fetal macrosomia, previous CS, and breech presentation.

Covariates were selected based on the review of existing literature: maternal sociodemographic background such as age, educational level or marital status [52–54], parity [55–57] and infant sex [58] have been considered known or suspected factors affecting child temperament.

### Data analyses

Since indications for CS and birth mode are causally related, placing both in one regression analysis could result in biased estimates due to the post-treatment bias [59–62]. We assessed the effects of delivery mode and indications for CS on ICQ scores by separate linear regressions (unadjusted and adjusted), comparing each type of CS to a vaginal delivery (or each type of indication to no indication). To further explore possible moderating effects of parity and infant’s sex on the association between the mode of birth and ICQ, we conducted multiple linear regressions with parity and infant sex interactions while controlling for the covariates listed above. Statistical significance was set at the alpha = 0.05 level. Statistical analysis was conducted using R [63].

## Results

### Sample description

The characteristics of the mothers and infants included in this study are summarized in Table 1. Of the 452 women who completed the ICQ at 6 weeks postpartum, 77% had vaginal birth, 12% planned CS for medical reasons, and 11% had undergone emergency CS. The numbers of female and male infants were approximately even (50,2 and 49,8%, respectively). A total of 43% women were primiparae. Most mothers (75%) were married, 22% were single, and 3% were divorced. The mean age was 31 years (range 18–45). The women who completed the ICQ at 9 months postpartum (n = 258) had similar characteristics except that the percentage of women with a high educational level increased slightly. No differences in infant sex, parity, maternal age or educational level were found by type of delivery.

**Table 1:** Characteristics of the sample.

	6 weeks postpartum (N = 452)	9 months postpartum (N = 258)
	N (%)	N (%)
Mode of delivery		
Vaginal	347 (76.8)	197 (76.4)
Planned CS for medical reasons	55 (12.2)	28 (10.9)
Emergency CS	50 (11.1)	33 (12.8)
Infant’s sex		
Boy	225 (49.8)	130 (50.4)
Girl	227 (50.2)	128 (49.6)
Parity		
Primipara	196 (43.4)	108 (41.9)
Multipara	256 (56.6)	150 (58.1)
Marital status		
Single	98 (21.7)	55 (21.3)
Married	338 (74.8)	194 (75.2)
Divorced	16 (3.5)	9 (3.5)
Educational level		
Low	41 (9.1)	20 (7.8)
Medium	244 (54.0)	129 (50.0)
High	167 (36.9)	109 (42.2)
	mean (SD)	mean (SD)
Maternal age (years)	30.61 (4.26)	30.81 (4.05)
ICQ scores	41.98 (10.08)	39.88 (10.51)
ICQ scores by mode of delivery		
Vaginal	41.82 (9.96)	39.44 (9.93)
Planned CS for medical reasons	42.78 (11.07)	40.82 (13.59)
Emergency CS	42.20 (9.93)	41.70 (11.03)
	N (%)	N (%)
Indications for CS		
Fetal hypoxia	37 (8.2)	25 (9.7)
Previous CS	32 (7.1)	18 (7.0)
Fetal macrosomia	15 (3.3)	9 (3.5)
Labor dysfunctions*	12 (2.7)	8 (3.1)
Breech presentation	22 (4.9)	12 (4.7)
Failure to progress	6 (1.3)	5 (1.9)

*Asynclitism and dystocia.

### Infant temperament at 6 weeks of age

In both the unadjusted (S2 Table) and adjusted (Table 2) analyses, children born by emergency or planned CS for medical reasons did not have significantly different ICQ scores at 6 weeks from those born by vaginal delivery. In the adjusted analysis, only marital status was significantly associated with ICQ scores, with single women rating their infants as less difficult than married women. The overall model fit was not significant.

**Table 2:** Association between the mode of delivery and infant temperament—Adjusted for sociodemographic factors, parity and infant’s sex.

	ICQ (6 weeks)			ICQ (9 months)		
	*B*	*SE*	*p-value*	*B*	*SE*	*p-value*
Intercept	43.07	1.11	< .001	39.22	1.54	< .001
Mode of delivery						
Planned CS for medical reasons	0.88	1.47	.550	1.05	2.13	.621
Emergency CS	0.44	1.53	.776	2.68	2.01	.184
Maternal age	-0.12	0.13	.361	-0.12	0.18	.526
Infant’s sex: boy	0.35	0.96	.711	-0.95	1.33	.477
Parity: multipara	-1.12	1.07	.297	0.79	1.45	.587
Educational level						
Low	0.65	1.72	.705	-2.14	2.54	.401
High	-0.02	1.03	.983	2.47	1.39	.077
Marital status						
Single	-3.39	1.25	.007	-2.07	1.74	.235
Divorced	-1.02	2.64	.699	-3.45	3.62	.341
Observations	452			258		
R^2^ / adj. R^2^	.020 / -.000			.043 /.008		
F-test	F(9, 442) = 1.00			F(9, 248) = 1.24		
	p = .44			p = .27		

Note: Default categories for dummy coded categorical predictors were set as follows: mode of delivery = vaginal; parity = primipara; educational level = medium; marital status = married; infant’s sex = girl. Maternal age was centered at the median (30 years).

**Fig 1:**
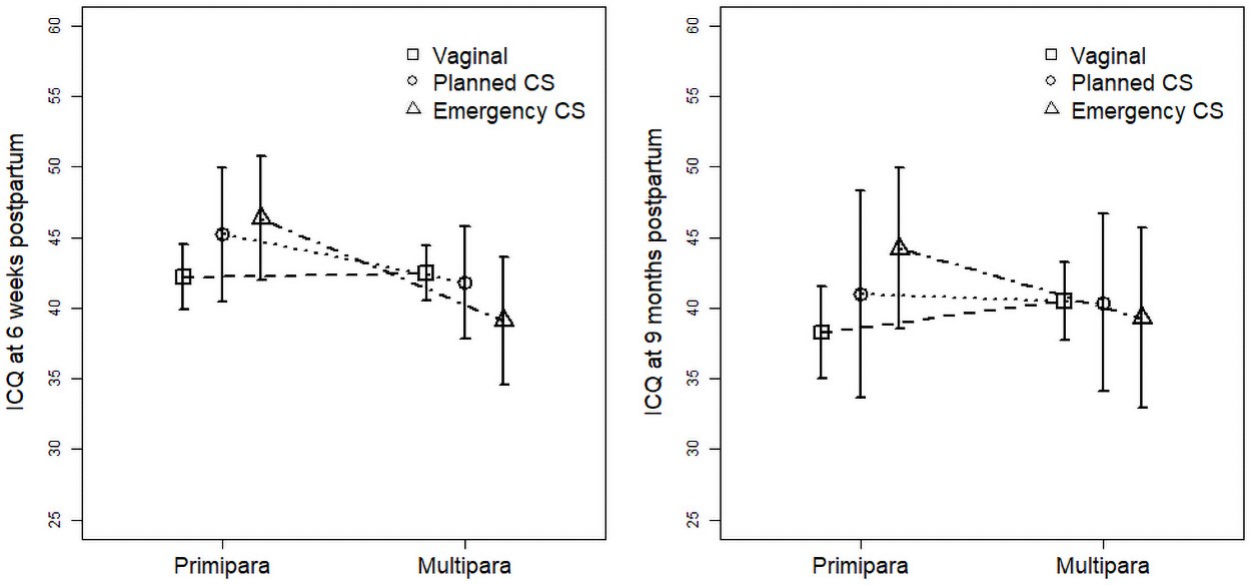
The effect of parity on the association between the mode of delivery and infant temperament at 6 weeks and 9 months postpartum.

The analysis of interactions suggested that parity moderates the effect of emergency CS on infant temperament ratings at 6 weeks postpartum (*B* = -7.57, *SE* = 3.07, *p* = 0.014) (Table 3; Fig 1) such that primiparae rated their infants’ temperament as more difficult if they had an emergency CS compared to vaginal birth, a finding that did not apply to multiparae. We found no significant interaction between birth mode and infant sex related to temperament ratings at age 6 weeks.

**Table 3:** Association between the mode of delivery and infant temperament—Interaction with parity.

	ICQ (6 weeks)			ICQ (9 months)		
	*B*	*SE*	*p-value*	*B*	*SE*	*p-value*
Intercept	42.18	1.16	< .001	38.28	1.64	< .001
Mode of delivery						
*Planned CS*	3.04	2.31	.188	2.72	3.38	.422
*Emergency CS*	4.19	2.15	.052	5.94	2.73	.030
Maternal age	-0.14	0.13	.268	-0.17	0.18	.363
Infant’s sex: boy	0.38	0.95	.688	-0.95	1.33	.477
Multipara	0.29	1.20	.807	2.22	1.67	.186
Educational level						
Low	0.54	1.71	.751	-2.30	2.55	.368
High	0.16	1.03	.880	2.65	1.39	.058
Marital Status						
Single	-3.28	1.25	.009	-1.83	1.74	.294
Divorced	-0.82	2.63	754	-3.20	3.61	.378
Planned CS x multipara	-3.71	2.99	.215	-2.82	4.35	.518
Emergency CS x multipara	-7.57	3.07	.014	-7.13	4.07	.081
Observations	452			258		
R^2^ / adj. R^2^	.035 /.011			.055 /.013		
F-statistics	F(11, 440) = 1.46			F(11, 246) = 1.31		
	p = .14			p = .22		

Note: Default categories for dummy coded categorical predictors were set as follows: mode of delivery = vaginal; parity = primipara; educational level = medium; marital status = married; infant’s sex = girl. Maternal age was centered at the median (30 years). At 6 weeks ppt: 22 primiparae gave birth per planned CS, 26 per emergency CS; 33 multiparae gave birth per planned CS, 24 per emergency CS. At 9 months ppt: 11 primiparae gave birth per planned CS, 19 per emergency CS; 17 multiparae gave birth per planned CS and 14 per emergency CS.

The unadjusted analysis did not show any significant associations between fetal hypoxia, previous CS, fetal macrosomia, breech presentation, failure to progress, or other labor dysfunctions and ICQ scores at 6 weeks (S3 Table). The results remained unchanged in the adjusted analysis (Table 4).

**Table 4:** Association between indications for cesarean section and infant temperament—Adjusted for sociodemographic factors, parity, and infant’s sex.

	ICQ (6 weeks)			ICQ (9 months)		
	*B*	*SE*	*p-value*	*B*	*SE*	*p-value*
Intercept	42.99	1.12	< .001	38.96	1.54	< .001
Fetal hypoxia	0.09	1.80	.959	0.43	2.37	.855
Previous CS	-0.15	2.00	.939	-0.17	2.73	.949
Fetal macrosomia	-0.30	2.80	.915	-2.11	3.80	.579
Labor dysfunctions*	-2.03	3.71	.585	0.01	5.01	.999
Breech presentation	2.71	2.31	.242	7.36	3.27	.025
Failure to progress	0.25	5.22	.961	3.44	6.27	.583
Maternal age	-0.12	0.13	.350	-0.12	0.18	.525
Infant’s sex: boy	0.56	0.97	.566	-0.52	1.34	.702
Multipara	-0.88	1.11	.427	1.09	1.51	.472
Educational level						
Low	0.67	1.73	.697	-1.67	2.57	.515
High	-0.12	1.04	.909	2.36	1.40	.092
Marital status						
Single	-3.52	1.27	.006	-2.20	1.77	.214
Divorced	-1.09	2.64	.680	-3.51	3.62	.332
Observations	452			258		
R^2^ / adj. R^2^	.023 / -.006			.058 /.008		
F-statistics	F(13, 438) = 0.8			F(13, 244) = 1.15		
	p = .66			p = .32		

Note: Maternal age was centered at the median (30 years), default categories for dummy coded categorical predictors were set as follows: parity = primipara, educational level = medium, marital status = married, infant’s sex = girl.

*Asynclitism and dystocia.

### Infant temperament at 9 months of age

In both the unadjusted (S2 Table) and adjusted (Table 2) analyses, neither emergency nor planned CS for medical reasons were significantly associated with difficult temperament 9 months postpartum. In the adjusted analysis, no variable was found to be significantly associated with the ICQ scores.

The analysis of interaction between parity and birth mode on infant temperament ratings at 9 months postpartum (Table 3; Fig 1) showed a similar pattern of results as at 6 weeks postpartum. Even though the moderating effect of parity was not significant for emergency CS (B = -7.13, SE = 4.07, p = 0.081), investigation of simple slopes indicated that primiparae rated their infants’ temperament less favorably if they had given birth per emergency CS compared to vaginal birth (*B* = 5.94, *SE* = 2.73, *p* = 0.030), whereas inverse but non-significant results were observed in multiparous women, who reported more difficult temperament if they had given birth vaginally compared to emergency CS (*B* = -1.20, *SE* = 2.99, *p* = 0.69). No significant interaction between birth mode and infant sex was found to affect infant temperament 9 months postpartum.

No indications for CS were found to be significantly associated with infant temperament at 9 months postpartum, except that breech presentation did predict a more difficult temperament (*B* = 7.61, *SE* = 3.21, *p* = 0.019); the overall model fit was not significant (S3 Table). The fully adjusted analysis yielded similar results (Table 4).

## Discussion and conclusions

In this study, we investigated the effects of cesarean section (CS) on difficult infant temperament assessed 6 weeks and 9 months postpartum. When considered in the absence of moderators, we found no association between either emergency or planned CS and infant temperament in our healthy sample. This null finding is consistent with several other studies, especially those that used large population-based samples and controlled for maternal health status in pregnancy. Such studies, too, reported no relation between CS and behavioral problems [6, 18, 19].

Our analyses suggested that primiparae rated their infants’ temperament as more difficult if they had an emergency CS compared to vaginal birth, a finding that did not apply to multiparae. However, the interaction of emergency CS and parity was significant only at 6 weeks. The fact that the interaction term for emergency CS and parity at 9 months postpartum was no longer significant may be due to a smaller sample size at 9 months compared to 6 weeks, which could have resulted in lower statistical power. Alternatively, it is possible that with time, primiparous women who gave birth through CS recovered from the stress related to emergency operative delivery, which resulted in less severe assessments of their children’s behavior.

Our finding that emergency CS may increase the risk of difficult temperament in infants born to first-time mothers, but not in those born to more experienced mothers, suggests that primiparae might be more vulnerable to the effects of emergency CS in terms of lower maternal self-esteem [64] and parenting competences [39], which may lead to higher levels of negative affectivity in their infants [45–47]. Multiparous women, on the other hand, have been found to have higher maternal self-efficacy and more optimal mother-child bonding [26–28], which enables them to interact with their infants more positively even in the aftermath of emergency CS. Relatedly, primiparous women tend to experience more anxiety in the postpartum period [26], which, combined with CS-related stress, may increase cortisol levels in maternal milk with potential effects on their infants’ temperament [65].

An alternate possibility is that these results may be confined to maternal perceptions of infant temperament, rather than (or in addition to) reflecting infants’ actual behaviors. Higher maternal self-efficacy in multiparous women [26, 27] may lead to their impressions of their infants being less open to influence from the experience of an unplanned medical procedure involving their newborn. In contrast, the ratings provided by first-time mothers may reflect their higher vulnerability to negative psychosocial impacts of CS including lower parenting self-esteem and higher anxiety [39, 64]. Moreover, higher anxiety in primiparous women who undergo CS may also be related to the fact that primiparae are more affected by CS in terms of their reproductive choices; existing studies show that delivery via CS is associated with a lower number of subsequent pregnancies [66].

Our finding that the effect of emergency CS on infant behavior may differ in primiparae and multiparae may help explain the inconsistent results of previous studies which evidenced both more positive [12], more negative [14], and equal [13] temperament ratings of infants born via CS in comparison to those born vaginally. Nevertheless, the effect of CS was not moderated by infant sex, which agrees with the results of previous studies [6, 16, 19].

Particular indications for CS had no effect on infant temperament ratings except for breech presentation that was associated with less favorable infant temperament 9 months postpartum. However, this finding is not conclusive as it only occurred 9 months and not 6 weeks postpartum and the overall model fit was not significant. Moreover, as we excluded women with serious health problems in pregnancy as well as seriously ill newborns, we explored only the effects of a limited range of indications. It is possible that other indications, especially those related to suboptimal health status in newborns, would predict infant temperamental difficulty. Indeed, Sirvinskiene et al. [15] observed that suboptimal newborn physiological functioning predicted emotional and behavioral problems at 1.5 year of age.

Investigation of the effects of CS on infant outcomes is methodologically challenging due to potentially confounding factors associated with both a higher risk of CS and infant behavioral outcomes. It is therefore a strength of our study that we accounted for such confounding by including only healthy mothers whose pregnancy resulted in the birth of a healthy newborn. Moreover, we excluded women who had CS performed on their own request without medical reasons. Those women may have a specific psychological profile which could be associated with behavioral characteristics of their children [67]. The strengths of our study also include differentiating between emergency and planned CS, taking into account the underlying indications for CS, examining infants’ temperament prospectively at two different time points, and considering parity as a potential moderator of effects.

Our study does, however, also have certain limitations. Its perhaps most important weakness is linked to the fact that infant temperament was rated by mothers, which obscures the degree to which the results reflect mother’s perceptions of their infant, or the infant’s behavior *per se*. The use of parent report to assess child temperament has been criticized with respect to issues of bias [68]. This concern may be particularly salient for the ICQ, in comparison to other temperament questionnaires, as it was explicitly designed to measure perceptions of “difficultness”. A related shortcoming is the lack of detail regarding temperament that is provided by the ICQ. More recently developed instruments, such as the Infant Behavior Questionnaire—Revised [69], provide a wider perspective on temperament, including dimensions associated with approach tendencies and capacities for behavioral control, to complement the emphasis on negative affectivity inherent to the concept of difficultness. Despite these critiques, however, questionnaires such as the ICQ are consistent with the literature, as, to best of our knowledge, all previous studies investigating the association between delivery mode and child temperament or behavioral problems relied on parental report [6, 12–16, 18, 19, 21]. Such frequent use of parent-administered questionnaires is due to the relative ease with which they can be employed, but also because they make use of parents’ vast experience with their children in contexts that are ethically or logistically difficult to measure in brief and artificial laboratory observations and require less interpretational inference than psychobiological measures of temperament [70]. Although bias is a real concern, contemporary temperament scholars contend that parent-report measures contain both subjective and objective components [71, 72], and parent-report measures have been shown to demonstrate superior predictive validity than laboratory observation indices [73]. To disentangle the degree to which CS has implications for temperament, future research should employ multiple methods.

Another limitation concerns sample attrition: although 1,190 mothers completed the questionnaire at baseline, only 661 completed the questionnaire at 6 weeks and 392 at 9 months postpartum. However, women who dropped out of the study differed from those who remained only with respect to educational level. Given the demographics of the Czech Republic, our sample was ethnically homogenous, including only White European population. Although this does not limit possible generalizations for the Czech population, it may limit the generalization potential of our findings to other countries or ethnically diverse populations.

In conclusion, we largely failed to find evidence linking surgical birth with adverse behavioral outcomes. An interaction between emergency CS and parity was found, suggesting that primiparous women rated their babies born by unexpected CS as more fussy, unadaptable, and unpredictable than infants born vaginally. However, based on our data, we cannot determine whether it is CS *per se* that affects firstborns’ behavior through biological pathways such as altered infant gut microbiota, or whether our results indicate that first-time mothers undergoing emergency surgical delivery tend to perceive their children’s behavior as more difficult. Given our null findings regarding the main effect of CS, it is yet premature to draw firm conclusions to inform clinical practice. Further research is needed to shed light on the potential risks associated with CS, preferably using observational methods for temperament assessment, investigating biological pathways through which may CS exert effects on children, while controlling carefully for indications for CS and other relevant confounders.

## Supporting information

S1 Table Differences between the women who dropped-out of the study from T2 to T3 and those who did not. (DOCX)

S2 Table Association between the mode of delivery and infant temperament—Unadjusted analyses. (DOCX)

S3 Table Association between indications for Caesarean section and infant temperament—Unadjusted analysis. (DOCX)
