# Supplementary material for: Parity moderates the effect of delivery mode on maternal ratings of infant temperament
Source: PLoS One. 2021 Aug 12;16(8):e0255367. doi: 10.1371/journal.pone.0255367 (PMC8360581; doi:10.1371/journal.pone.0255367)
Supplement: S1 Table — (DOCX) [file pone.0255367.s001.docx]

**S1 Table. Differences between the women who dropped-out of the study from T2 to T3 and those who did not**

|  |  | Differences between  T2 and T3 samples | | |  |
| --- | --- | --- | --- | --- | --- |
|  |  | *OR* | *CI* | *p-value* |  |
| Intercept |  | 1.12 | 0.72 – 1.76 | 0.609 |  |
| Mode of delivery |  | | | |  |
| Planned CS |  | 2.09 | 0.84 – 5.50 | 0.121 |  |
| Emergency CS |  | 1.27 | 0.37 – 4.35 | 0.700 |  |
| Maternal age |  | 0.98 | 0.93 – 1.03 | 0.443 |  |
| Infant’s sex: boy |  | 0.90 | 0.61 – 1.32 | 0.597 |  |
| Multipara |  | 0.93 | 0.60 – 1.44 | 0.731 |  |
| Educational level |  | | | |  |
| Low |  | 1.05 | 0.53 – 2.07 | 0.887 |  |
| High |  | 0.66 | 0.43 – 1.00 | **0.049** |  |
| Marital Status |  | | | |  |
| Single |  | 0.90 | 0.54 – 1.48 | 0.666 |  |
| Divorced |  | 1.10 | 0.38 – 3.13 | 0.863 |  |
| Fetal hypoxia |  | 0.56 | 0.17 – 1.86 | 0.336 |  |
| Previous CS |  | 0.72 | 0.24 – 2.07 | 0.547 |  |
| Fetal macrosomia |  | 0.70 | 0.20 – 2.32 | 0.560 |  |
| Labor dysfunctions* |  | 0.97 | 0.17 – 5.25 | 0.975 |  |
| Breech presentation |  | 0.64 | 0.20 – 1.94 | 0.434 |  |
| Failure to progress |  | 0.28 | 0.01 – 2.70 | 0.311 |  |
| Observations |  | 452 | | |  |

*Asynclitism and dystocia

Multiple logistic regression. The dependent variable is whether the subject dropped out or not. OR > 1 indicates a higher probability of drop-out, while OR < 1 lower probability of drop-out.

Note: Maternal age was centered at the median (30 years), default categories for dummy coded categorical predictors were set as follows: mode of delivery = vaginal, parity = primipara, education = medium, marital status = married, infant’s sex = girl.
