# Supplementary material for: Parity moderates the effect of delivery mode on maternal ratings of infant temperament
Source: PLoS One. 2021 Aug 12;16(8):e0255367. doi: 10.1371/journal.pone.0255367 (PMC8360581; doi:10.1371/journal.pone.0255367)
Supplement: S2 Table — (DOCX) [file pone.0255367.s002.docx]

**S2 Table. Association between the mode of delivery and infant temperament – unadjusted analyses**

|  |  | ICQ (6 weeks) | | |  | ICQ (9 months) | | |
| --- | --- | --- | --- | --- | --- | --- | --- | --- |
|  |  | *B* | *SE* | *p-value* |  | *B* | *SE* | *p-value* |
| Intercept |  | -0.02 | 0.05 | .769 |  | -0.04 | 0.07 | .559 |
| Mode of delivery | | | | | | | | |
| *Planned CS* |  | 0.10 | 0.15 | .511 |  | 0.13 | 0.20 | .516 |
| *Emergency CS* |  | 0.04 | 0.15 | .803 |  | 0.21 | 0.19 | .255 |
| Observations |  | 452 | | |  | 258 | | |
| R^2^ / adj. R^2^ |  | .001 / -.003 | | |  | .006 / -.002 | | |
| F-test |  | F(2, 449) = 0.23  p = .79 | | |  | F(2, 255) = 0.78  p = .46 | | |
