# Supplementary material for: Parity moderates the effect of delivery mode on maternal ratings of infant temperament
Source: PLoS One. 2021 Aug 12;16(8):e0255367. doi: 10.1371/journal.pone.0255367 (PMC8360581; doi:10.1371/journal.pone.0255367)
Supplement: S3 Table — (DOCX) [file pone.0255367.s003.docx]

**S3 Table. Association between indications for Caesarean section and infant temperament – unadjusted analysis**

|  |  | ICQ (6 weeks) | | |  | ICQ (9 months) | | |
| --- | --- | --- | --- | --- | --- | --- | --- | --- |
|  |  | *B* | *SE* | *p-value* |  | *B* | *SE* | *p-value* |
| Intercept |  | 41.89 | 0.53 | <.001 |  | 39.52 | 0.72 | <.001 |
| Fetal hypoxia |  | -0.20 | 1.79 | .912 |  | -0.37 | 2.33 | .875 |
| Previous CS |  | 0.05 | 1.94 | .978 |  | 0.67 | 2.63 | .798 |
| Fetal macrosomia |  | 0.00 | 2.78 | 1.00 |  | -1.85 | 3.78 | .626 |
| Breech presentation |  | 2.74 | 2.24 | .222 |  | 7.61 | 3.21 | .019 |
| Failure to progress |  | -0.13 | 5.22 | .980 |  | 4.22 | 6.24 | .499 |
| Other labor dysfunctions* |  | -1.27 | 3.70 | .732 |  | -0.65 | 4.94 | .896 |
| Observations |  | 452 | | |  | 258 | | |
| R^2^ / adj. R^2^ |  | .004 / -.009 | | |  | .024 / .001 | | |
| F-test |  | F(6, 445) = 0.30  p = .94 | | |  | F(6, 251) = 1.03  p = .41 | | |

*Asynclitism and dystocia
